# Associations of artificially sweetened beverage intake with disease recurrence and mortality in stage III colon cancer: Results from CALGB 89803 (Alliance)

**DOI:** 10.1371/journal.pone.0199244

**Published:** 2018-07-19

**Authors:** Brendan J. Guercio, Sui Zhang, Donna Niedzwiecki, Yanping Li, Ana Babic, Vicente Morales-Oyarvide, Leonard B. Saltz, Robert J. Mayer, Rex B. Mowat, Renaud Whittom, Alexander Hantel, Al Benson, Daniel Atienza, Michael Messino, Hedy Kindler, Alan Venook, Shuji Ogino, Emilie S. Zoltick, Meir Stampfer, Kimmie Ng, Kana Wu, Walter C. Willett, Edward L. Giovannucci, Jeffrey A. Meyerhardt, Charles S. Fuchs

**Affiliations:** 1 Department of Medicine, Brigham and Women’s Hospital, Boston, Massachusetts, United States of America; 2 Dana-Farber/Partners CancerCare, Boston, Massachusetts, United States of America; 3 Alliance Statistics and Data Center, Duke University, Durham, North Carolina, United States of America; 4 Department of Nutrition, Harvard T.H. Chan School of Public Health, Boston, Massachusetts, United States of America; 5 Memorial Sloan Kettering Cancer Center, New York, New York, United States of America; 6 Toledo Community Hospital Oncology Program, Toledo, Ohio, United States of America; 7 Hôpital du Sacré-Coeur de Montréal, Montreal, Canada; 8 Edward-Elmhurst Healthcare, Naperville, Illinois, United States of America; 9 Robert H. Lurie Comprehensive Cancer Center, Northwestern University, Chicago, Illinois, United States of America; 10 Virginia Oncology Associates, Norfolk, Virginia, United States of America; 11 Southeast Clinical Oncology Research (SCOR) Consortium, Mission Hospitals, Incorporated, Asheville, North Carolina, United States of America; 12 University of Chicago Comprehensive Cancer Center, Chicago, Illinois, United States of America; 13 University of California at San Francisco Comprehensive Cancer Center, San Francisco, California, United States of America; 14 Department of Epidemiology, Harvard T.H. Chan School of Public Health, Boston, Massachusetts, United States of America; 15 Division of Molecular Pathological Epidemiology (MPE), Department of Pathology, Brigham and Women’s Hospital, Boston, Massachusetts, United States of America; 16 Department of Pathology, Harvard Medical School, Boston, Massachusetts, United States of America; 17 Section of Preventive Medicine and Epidemiology, Department of Medicine, Boston University School of Medicine, Boston, Massachusetts, United States of America; 18 Division of Genetics, Department of Medicine, Brigham and Women’s Hospital, Boston, Massachusetts, United States of America; 19 Channing Division of Network Medicine, Department of Medicine, Brigham and Women’s Hospital, Harvard Medical School, Boston, Massachusetts, United States of America; 20 Yale Cancer Center, Yale School of Medicine, New Haven, Connecticut, United States of America; Hospital de Santa Maria, PORTUGAL

## Abstract

**Purpose:**

Observational studies have demonstrated increased colon cancer recurrence and mortality in states of excess energy balance, as denoted by factors including sedentary lifestyle, diabetes, increased dietary glycemic load, and increased intake of sugar-sweetened beverages. Nonetheless, the relation between artificially sweetened beverages, a popular alternative for sugar-sweetened beverages, and colon cancer recurrence and survival is unknown.

**Methods:**

We analyzed data from 1,018 patients with stage III colon cancer who prospectively reported dietary intake during and after chemotherapy while enrolled in a National Cancer Institute-sponsored trial of adjuvant chemotherapy. Using Cox proportional hazards regressions, we assessed associations of artificially sweetened beverage intake with cancer recurrence and mortality.

**Results:**

Patients consuming one or more 12-ounce servings of artificially sweetened beverages per day experienced an adjusted hazard ratio for cancer recurrence or mortality of 0.54 (95% confidence interval, 0.36 to 0.80) when compared to those who largely abstained (*P*_trend_ = .004). Similarly, increasing artificially sweetened beverage intake was also associated with a significant improvement in both recurrence-free survival (*P*_trend_ = .005) and overall survival (*P*_trend_ = .02). Substitution models demonstrated that replacing a 12-ounce serving of a sugar-sweetened beverage with an isovolumetric serving of an artificially sweetened beverage per day was associated with a 23% lower risk of cancer recurrence and mortality (relative risk, 0.77; 95% confidence interval, 0.63 to 0.95; *P* = .02).

**Conclusion:**

Higher artificially sweetened beverage consumption may be associated with significantly reduced cancer recurrence and death in patients with stage III colon cancer. This association may be mediated by substitution for sugar-sweetened alternatives. Further studies are needed to confirm these findings.

## Introduction

A growing body of literature supports an association between excess energy balance—as denoted by sedentary lifestyle, type 2 diabetes, increased dietary glycemic load, elevated serum C-peptide, and Western pattern diet—and inferior survival for patients with colon cancer [1–7]. Sugar-sweetened beverage intake has been linked with increased risk of obesity, type 2 diabetes, and related cardio-metabolic disease [8–14], and also increased cancer recurrence and mortality in stage III colon cancer patients [15].

Artificially sweetened beverages serve as low-calorie substitutes for sugar-sweetened beverages, potentially reducing excess energy balance. Nonetheless, concerns that artificial sweeteners may increase incidence of obesity, diabetes, and cancer have been raised [16–19]. The foundation for such concern is uncertain, as studies on the impact of artificially sweetened beverages on weight gain, diabetes, and cardio-metabolic disease have produced mixed results [14, 20–29]. Furthermore, while selected studies in animal models suggest increased incidence of certain cancers with artificial sweetener exposure [30, 31], epidemiologic studies in humans have not demonstrated such relationships [32–35].

Given existing uncertainties regarding the health effects of artificially sweetened beverages, we conducted a prospective cohort study nested in a National Cancer Institute (NCI)-sponsored randomized clinical trial of adjuvant therapy for stage III colon cancer examining associations of artificially sweetened beverage intake with colon cancer recurrence and mortality. Measurement of artificially sweetened beverage intake was conducted prior to any documentation of cancer recurrence. Careful and comprehensive documentation during the trial of patient performance status, pathologic stage, post-operative treatment, and diet and lifestyle habits allowed concurrent effects of patient, disease, and treatment characteristics to be examined.

## Methods

### Study population

Patients in this cohort participated in the Cancer and Leukemia Group B (CALGB, now part of Alliance for Clinical Trials in Oncology) 89803 adjuvant therapy trial for stage III colon cancer, comparing weekly 5-fluorouracil and leucovorin to weekly irinotecan, 5-fluorouracil, and leucovorin (ClinicalTrials.gov NCT00003835) [36]. Between April 1999 and May 2001, 1,264 patients were enrolled in the trial. After 87 patients enrolled, an amendment to the protocol required patients to complete a self-administered questionnaire capturing diet and lifestyle habits midway through therapy (4 months after surgery; questionnaire 1 [Q1]) and again 6 months after completion of treatment [14 months after surgery; Q2]).

**Fig 1:**
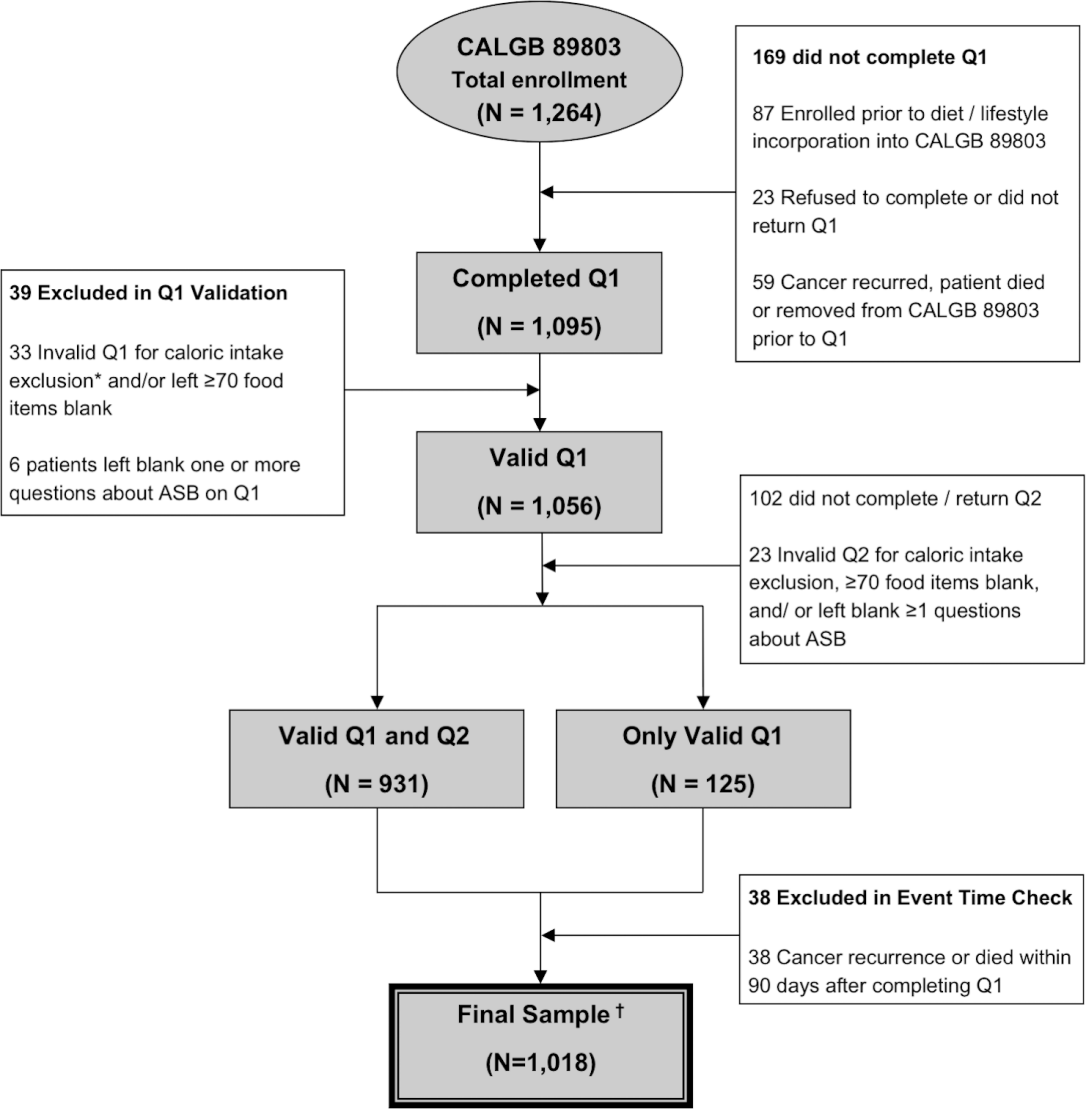
Derivation of the study cohort. Abbreviations: ASB, artificially sweetened beverages; CAGLB, Cancer and Leukemia Group B; Q1, questionnaire 1 (midway through adjuvant therapy); Q2, questionnaire 2 (6 months after completion of adjuvant therapy). *Caloric intake exclusion = Less than 600 calories or greater than 4,200 calories per day for men and less than 500 calories or greater than 3,500 calories per day for women. † 6 patients in the final sample are missing physical activity in questionnaire 1 and 3 are missing physical activity in questionnaire 2. 1 patient is missing body mass index in questionnaire 1 and 1 is missing body mass index in questionnaire 2.

Eligibility required complete surgical resection within 56 days prior to trial entry, no prior chemotherapy or radiation therapy for treatment of the tumor, regional lymph node metastases without distant metastases, a baseline Eastern Cooperative Oncology Group performance status of 0 to 2 [37], and adequate bone marrow, renal, and hepatic function. Patients were excluded if they reported significantly implausible caloric intake (<600 or >4,200 calories per day for men; <500 or >3,500 calories per day for women), left >70 food items blank, or left blank one or more questions about artificially sweetened beverages on Q1 or Q2. Finally, to avoid biases due to declining health immediately before cancer recurrence or death, we excluded patients who experienced cancer recurrence or death within 90 days following Q1 completion. **Fig 1** demonstrates derivation of the final 1,018 patient cohort. There were no appreciable differences in baseline characteristics between patients eligible for dietary analysis and the remaining patients enrolled in CALGB 89803 [2]. All patients signed study-specific informed consent approved by the NCI Cancer Treatment Evaluation Program and each participating site’s institutional review board (IRB), including the Dana-Farber/Harvard Cancer Center IRB.

### Dietary assessment

Patients completed validated semi-quantitative food frequency questionnaires (FFQs) [38, 39]. Participants were asked how often, on average over the previous 3 months, they consumed a specific food portion size, with up to nine possible responses ranging from never to six or more per day. We computed nutrient intakes by multiplying the frequency of consumption of each food by the nutrient content of the specified portions using composition values from the Department of Agriculture supplemented with other data [40]. Nutrient values were energy-adjusted using the residuals method [41].

On each FFQ, patients reported their intake of a standard 355-mL (12-ounce [oz.]) serving (1 glass, can, or bottle) of each of the following low calorie, artificially sweetened beverages: caffeinated colas, caffeine-free colas, and other carbonated beverages (e.g., diet ginger ale). We summed consumption of these beverages as total artificially sweetened beverage intake. In other cohorts, we previously assessed the reproducibility of our FFQ in measuring consumption of beverages. Correcting for within-person variation, the Pearson correlation coefficient between the FFQ and an independent collection of dietary records was 0.84 for cola and 0.36–0.40 for non-cola carbonated beverages [42]. We separately validated this FFQ among cancer patients receiving chemotherapy [43].

Patients who completed Q1 without cancer recurrence prior to Q1 completion were included in these analyses. Median time from study entry to Q1 was 3.5 months (95% range, 2.5 to 5.0 months; full range, 0.2 to 9.9 months). We updated dietary exposures on the basis of results of Q2 using cumulative averaging as previously described [4], but weighted proportional to times between Q1 and Q2 and then between Q2 and disease-free survival (DFS) time. For example, if a patient completed Q1 at 4 months, completed Q2 at 14 months, and had a cancer recurrence at 30 months, the total time between Q1 and cancer recurrence was 26 months; 38% of that time was between Q1 and Q2 and 62% of that time was between Q2 and the recurrence.

### Tumor assessments for *KRAS*, *BRAF*, *PIK3CA*, and *TP53* mutations, microsatellite instability (MSI), and *PTGS2* expression

Polymerase chain reaction (PCR) and pyrosequencing targeted for mutation hotspots in *PIK3CA* exons 9 and 20 [44], *BRAF* codon 600 [45], and *KRAS* codons 12 and 13 were performed, as previously described [46, 47]. Mutations in *TP53* exons 5 to 8 were determined by Sanger sequencing and sequencing by hybridization, as previously described [48]. MSI was assessed by PCR for 10 microsatellite markers; tumors with instability in ≥50% of the loci were classified as MSI-high; for 28 cases without PCR MSI results, those with loss of *MLH1* or *MSH2* assessed by immunohistochemistry were classified as MSI-high [49]. *PTGS2* expression level was assessed by immunohistochemistry as previously described [50].

### Study endpoints

The primary endpoint of our study was DFS, defined as time from completion of Q1 to tumor recurrence, occurrence of a new primary colon tumor, or death from any cause. We also assessed recurrence-free survival (RFS), defined as time from completion of Q1 to tumor recurrence or occurrence of a new primary colon tumor. For RFS, patients who died without known tumor recurrence were censored at last documented physician evaluation. Finally, overall survival (OS) was defined as time from completion of Q1 to death from any cause.

### Statistical analysis

In the clinical trial, there was no statistically significant difference in either DFS or OS between treatment arms [36]. Therefore, data for patients in both arms were combined and analyzed according to frequency categories of artificially sweetened beverage intake. For the primary analysis, artificially sweetened beverage intake was classified into five frequency categories (< 2 servings per month, 2 per month to 2 per week, 3–6 per week, 1 to < 2 per day, ≥ 2 per day), consistent with prior analyses of beverage intake in this cohort [15].

Cox proportional hazards regression was used to determine the simultaneous impact of other variables potentially associated with each outcome [51]. As previously described [41], we used time-varying covariates to adjust for total calories, physical activity [52], and body mass index (BMI). Other covariates, including sex, age, depth of invasion through bowel wall, number of positive lymph nodes, baseline performance status, chemotherapy treatment group, and aspirin use were entered into the model as fixed covariates. In secondary multivariable analyses, we further adjusted for time-varying Western and prudent pattern diets (as defined previously[4]), dietary glycemic load, cereal fiber consumption, and intake of coffee, caffeine, sugar-sweetened beverages, and total fluid intake (a sum of intake of water, tea, juice, milk, coffee, and sugar-sweetened beverages). We included indicator variables for missing values in the multivariate models.

We tested for linear trends across frequency categories of intake by assigning each participant the median value for each frequency category and modeling the value as a continuous variable, consistent with previous studies [2, 4, 15, 53]. The proportionality of hazards assumption for the effect of artificially sweetened beverages and the difference in intake between artificially sweetened beverages and sugar-sweetened beverages was satisfied by examining each as a time-dependent covariate in the model. In subgroup exploratory analyses on the effect of artificially sweetened beverage consumption, we collapsed intake into four categories to conserve power, creating the following groupings: <2 servings per month, 2 per month to 2 per week, 3–6 per week, and ≥1 per day. In subgroup exploratory analyses, tests for interaction between artificially sweetened beverage intake and each covariate were performed by including the cross product of ordinal artificially sweetened beverage intake categories and each dichotomized covariate in a Cox proportional hazards regression model.

To evaluate the effect of substituting artificially sweetened beverages for sugar-sweetened beverages, we built isovolumetric substitution models wherein we treated intake as a continuous variable and used Cox proportional hazards regression to calculate the difference in coefficients between intake of artificially sweetened and sugar-sweetened beverages, similar to prior substitution analyses [54]. The coefficients from these models can be interpreted as the estimated effect of substituting a certain volume of artificially sweetened beverage for an equivalent volume of sugar-sweetened beverage. These multivariate models were adjusted for known and potential predictors of patient outcome including age (continuous variable in years), sex (male or female), depth of invasion through bowel wall (binary variable, pT1-2 or pT3-4), number of positive lymph nodes (binary variable, 1–3 nodes or ≥4 nodes), baseline performance status (binary variable, 0 or 1–2), chemotherapy treatment group (binary variable, 5-fluorouracil and leucovorin or irinotecan, 5-fluorouracil, and leucovorin), consistent aspirin use (yes on Q1 and Q2), and time-varying total caloric intake (continuous variable, in kilocalories per day), physical activity (continuous variable in metabolic equivalent task hours per week), and body mass index (continuous variable in kilogram per meter-squared).

Analyses were performed with SAS version 9.4 (SAS Institute, Cary, NC). *P* values are 2-sided and not adjusted for multiple comparisons. A *P* value less than .05 was considered statistically significant. Data collection was conducted by the Alliance Statistics and Data Center. Data quality was ensured through review by the Alliance Statistics and Data Center and by the study chairperson following Alliance policies. All log files of the statistical analyses can be found in the supplemental data section. Analyses were based on the study database frozen on November 9, 2009.

## Results

### Baseline characteristics

Baseline characteristics by frequency of artificially sweetened beverage consumption are displayed in **Table 1**. Frequent drinkers of artificially sweetened beverages were younger and had higher BMI and greater total caloric intake. They were more likely to adhere to a Western dietary pattern and use aspirin regularly, but less likely to be physically active or consume coffee or sugar-sweetened beverages. They tended to have less tumor invasion through the bowel wall and fewer positive lymph nodes.

**Table 1:** Baseline characteristics by artificially sweetened beverage intake (n = 1,018, median follow-up = 7.3 years).

No. Deaths and Total	Artificially Sweetened Beverage Intake (12 oz. servings)				
	<2/mo	2/mo-2/wk	3-6/wk	1 to <2/d	≥2/d
	161/483	77/265	47/156	14/72	10/42
Median (range)	0.0 (0.0–0.1)	0.2 (0.1–0.4)	0.6 (0.4–1.0)	1.4 (1.0–2.0)	2.5 (2.1–7.9)
Male sex, No. (%)	267 (55.3)	143 (54.0)	92 (59.0)	46 (63.9)	26 (61.9)
Age, years, median (range)	59 (21–85)	63.0 (24–81)	63 (34–80)	59 (27–77)	53 (28–75)
Race, No. (%)					
White	422 (87.4)	239 (90.2)	142 (91.0)	62 (86.1)	41 (97.6)
Black	34 (7.0)	17 (6.4)	7 (4.5)	7 (9.7)	1 (2.4)
Other	27 (5.6)	9 (3.4)	7 (4.5)	3 (4.2)	0 (0.0)
Baseline performance status, No. (%)^a^					
ECOG PS 0	354 (73.3)	195 (73.6)	118 (75.6)	46 (63.9)	33 (78.6)
ECOG PS 1, 2	119 (24.6)	68 (25.7)	31 (19.9)	24 (33.3)	9 (21.4)
Status unknown	10 (2.1)	2 (0.8)	7 (4.5)	2 (2.8)	0 (0.0)
pT Stage, No. (%)					
pT1,2	66 (13.7)	24 (9.1)	23 (14.7)	14 (19.4)	10 (23.8)
pT3,4	406 (84.1)	238 (89.8)	127 (81.4)	55 (76.4)	31 (73.8)
Missing	11 (2.3)	3 (1.1)	6 (3.8)	3 (4.2)	1 (2.4)
Positive lymph nodes, No. (%)					
1–3	284 (58.8)	178 (67.2)	105 (67.3)	43 (59.7)	31 (73.8)
≥4	189 (39.1)	85 (32.1)	46 (29.5)	27 (37.5)	11 (26.2)
Nodes unknown	10 (2.1)	2 (0.8)	5 (3.2)	2 (2.8)	0 (0.0)
Tumor differentiation, No. (%)					
Well/moderate	352 (72.9)	211 (79.6)	113 (72.4)	57 (79.2)	32 (76.2)
Poor/undifferentiated	119 (24.6)	52 (19.6)	38 (24.4)	13 (18.1)	10 (23.8)
Missing	12 (2.5)	2 (0.8)	5 (3.2)	2 (2.8)	0 (0.0)
Clinical bowel obstruction at presentation, No. (%)	109 (22.6)	61 (23.0)	36 (23.1)	10 (13.9)	6 (14.3)
Bowel perforation at presentation, No. (%)	19 (3.9)	8 (3.0)	11 (7.1)	3 (4.2)	3 (7.1)
Treatment arm, No. (%)					
FU/LV	244 (50.5)	136 (51.3)	85 (54.5)	33 (45.8)	15 (35.7)
IFL	239 (49.5)	129 (48.7)	71 (45.5)	39 (54.2)	27 (64.3)
Smoking status^b^					
Never	225 (46.6)	119 (44.9)	60 (38.5)	28 (38.9)	23 (54.8)
Ever	258 (53.4)	146 (55.1)	96 (61.5)	44 (61.1)	19 (45.2)
BMI, kg/m^2^, median (range)^b^	26.3 (15.7–49.9)	27.4 (17.2–51.8)	28.0 (17.5–49.9)	29.3 (16.3–46.2)	30.3 (19.5–46.3)
Physical activity, MET h/w, median (range)^b^	5.4 (0.0–147.4)	4.6 (0.0–119.9)	4.9 (0.0–77.9)	3.2 (0.0–60.4)	3.3 (0.0–114.2)
Aspirin user, No. (%)^b^	112 (23.2)	75 (28.3)	50 (32.1)	31 (43.1)	12 (28.6)
Multivitamin user, No. (%)^b^	213 (44.1)	142 (53.6)	86 (55.1)	38 (52.8)	23 (54.8)
Dietary intake, median (interquartile range) ^b^					
Total energy intake, kcal/d	2046 (1564–2553)	1874 (1458–2258)	1878 (1490–2230)	2031 (1474-2337-)	2316 (1715–2779)
Coffee intake, serving/d	0.9 (0.0–2.5)	1.0 (0.1–2.5)	1.0 (0.1–2.5)	0.8 (0.0–2.5)	0.1 (0.0–2.5)
Caffeine intake, mg/d	122.2 (41.0–290.1)	123.7 (31.8–263.1)	102.5 (32.8–237.1)	101.6 (44.3–280.1)	138.3 (99.4–274.7)
Alcohol intake, g/d	0.3 (0.0–4.6)	0.5 (0.0–3.5)	0.3 (0.0–4.9)	0.0 (0.0–2.6)	0.0 (0.0–1.8)
Sugar-sweetened beverages, serving/d	0.5 (0.1–1.0)	0.2 (0.1–0.6)	0.2 (0.0–0.7)	0.2 (0.1–0.9)	0.2 (0.0–1.3)
Dietary glycemic load, No. ≥ median (%)^b^	244 (50.5)	142 (53.6)	77 (49.4)	27 (37.5)	19 (45.2)
Western dietary pattern, No. ≥ median (%)^b^	258 (53.4)	121 (45.7)	66 (42.3)	37 (51.4)	27 (64.3)
Prudent dietary pattern, No. ≥ median (%)^b^	237 (49.1)	129 (48.7)	82 (52.6)	34 (47.2)	27 (64.3)

BMI kg/m^2^, body mass index in kilograms per meter-squared; d, day; ECOG PS, Eastern Cooperative Oncology Group Performance Status; FU/LV, fluorouracil and leucovorin; g, grams; IFL, irinotecan, fluorouracil, leucovorin; kcal, kilocalories; MET h/w, metabolic equivalent task hours per week; mg, milligrams; mo, month; No., number; oz., ounce; T stage, tumor stage; wk, week

^a^Baseline performance status (PS): PS 0 = fully active; PS 1 = restricted in physically strenuous activity but ambulatory and able to carry out light work; PS 2 = ambulatory and capable of all self-care but unable to carry out any work activities, up and about more than 50% of waking hours.

^b^As reported on questionnaire 1

### Associations of artificially sweetened beverage intake with cancer recurrence and death

Median follow-up from Q1 completion was 7.3 years. During follow-up, 348 of the 1,018 patients in the final cohort experienced colon cancer recurrence or developed new primary tumors; 265 of these patients died. An additional 44 patients died without documented recurrence.

Our predefined primary endpoint was DFS. Increasing artificially sweetened beverage intake was associated with significantly lower risk of cancer recurrence or mortality after adjusting for other predictors of cancer recurrence (**Table 2**). Compared with patients who largely abstained from artificially sweetened beverage intake, patients who consumed 2 or more 12 oz. servings per day experienced an adjusted hazard ratio (HR) for disease recurrence or mortality of 0.58 (95% confidence interval [CI], 0.31 to 1.07; *P*_trend_ = .004). Recognizing a limited number of patients reported consumption in this highest category of intake (n = 42), we repeated the analysis, collapsing the highest two categories, and found that patients reporting 1 or more 12 oz. servings per day experienced an adjusted HR of 0.54 (95% CI 0.36 to 0.80). Similarly, increasing artificially sweetened beverage intake was associated with a significant improvement in both RFS (*P*_trend_ = .005) and OS (*P*_trend_ = .02). These results were largely unchanged when further adjusted for time-varying Western and prudent dietary patterns, dietary glycemic load, cereal fiber consumption, and intake of sugar-sweetened beverages and coffee (DFS, *P*_trend_ = .005; RFS, *P*_trend_ = .007; OS, *P*_trend_ = .02). Moreover, the results were unchanged after adjusting for total caffeine intake (DFS, *P*_trend_ = .005; RFS, *P*_trend_ = .007; OS, *P*_trend_ = .02) and total fluid intake (DFS, *P*_trend_ = .004; RFS, *P*_trend_ = .006; OS, *P*_trend_ = .01).

**Table 2:** Associations between artificially sweetened beverage intake and colon cancer recurrence and mortality.

	Categories of artificially sweetened beverage intake (12 oz. servings)					
	< 2/mo	2/mo-2/wk	3-6/wk	1 to < 2/d	≥ 2/d	*P*_trend_^a^
**Disease-free survival**						
Events and total	194/483	109/265	59/156	19/72	11/42	.
HR (95% CI), Adjusted 1	1.0	1.00 (0.79–1.26)	0.89 (0.66–1.19)	0.56 (0.35–0.89)	0.57 (0.31–1.04)	0.004
HR (95% CI), Adjusted 2	1.0	0.94 (0.74–1.20)	0.89 (0.66–1.20)	0.52 (0.32–0.84)	0.58 (0.31–1.07)	0.004
				0.54 (0.36–0.80)		
**Recurrence-free survival**						
Events and total	175/483	97/265	50/156	16/72	10/42	.
HR (95% CI), Adjusted 1	1.0	0.98 (0.77–1.26)	0.84 (0.61–1.15)	0.53 (0.32–0.88)	0.58 (0.31–1.10)	0.005
HR (95% CI), Adjusted 2	1.0	0.95 (0.73–1.23)	0.86 (0.62–1.18)	0.50 (0.30–0.84)	0.58 (0.30–1.10)	0.005
				0.53 (0.35–0.81)		
**Overall survival**						
Events and total	161/483	77/265	47/156	14/72	10/42	.
HR (95% CI), Adjusted 1	1.0	0.83 (0.63–1.10)	0.84 (0.60–1.16)	0.51 (0.29–0.87)	0.63 (0.33–1.20)	0.02
HR (95% CI), Adjusted 2	1.0	0.76 (0.58–1.01)	0.82 (0.59–1.14)	0.46 (0.26–0.79)	0.65 (0.34–1.23)	0.02
				0.52 (0.34–0.81)		

Adjusted 1, adjusted for time-varying total calorie intake (continuous variable in kilocalories per day).

Adjusted 2, further adjusted for age (continuous variable in years), sex (male or female), depth of invasion through bowel wall (binary variable, pT1-2 or pT3-4), number of positive lymph nodes (binary variable, 1–3 nodes or ≥4 nodes), baseline performance status (binary variable, 0 or 1–2), chemotherapy treatment group (binary variable, 5-fluorouracil and leucovorin or irinotecan, 5-fluorouracil, and leucovorin), consistent aspirin use (yes on Q1 and Q2), and time-varying physical activity (continuous variable in metabolic equivalent task hours per week), and body mass index (continuous variable in kilogram per meter-squared).

Abbreviations: CI, confidence interval; HR, hazard ratio; d, day; mo, month; oz., ounce; Q1, questionnaire 1, midway through adjuvant chemotherapy; Q2, questionnaire 2, 14 months after surgery; wk, week

^a^Two-sided *P* value. Trend across five consumption levels.

As described in Methods, we excluded patients who developed cancer recurrence or died within 90 days of completing Q1 in our primary analyses to address the possibility that changes in dietary habits could reflect occult cancer or impending death. To further address this issue, we repeated the Cox proportional hazard models excluding patients who developed recurrence or died within 180 days of Q1 completion (n = 974) as a sensitivity analysis, and our results remained largely unchanged. Individuals reporting ≥1 artificially sweetened beverages per day continued to demonstrate a reduction in risk of mortality or disease recurrence compared to individuals drinking <2 per month (HR, 0.60; 95% CI, 0.40 to 0.91; *P*_trend_ = .01).

### Stratified analyses

**Fig 2:**
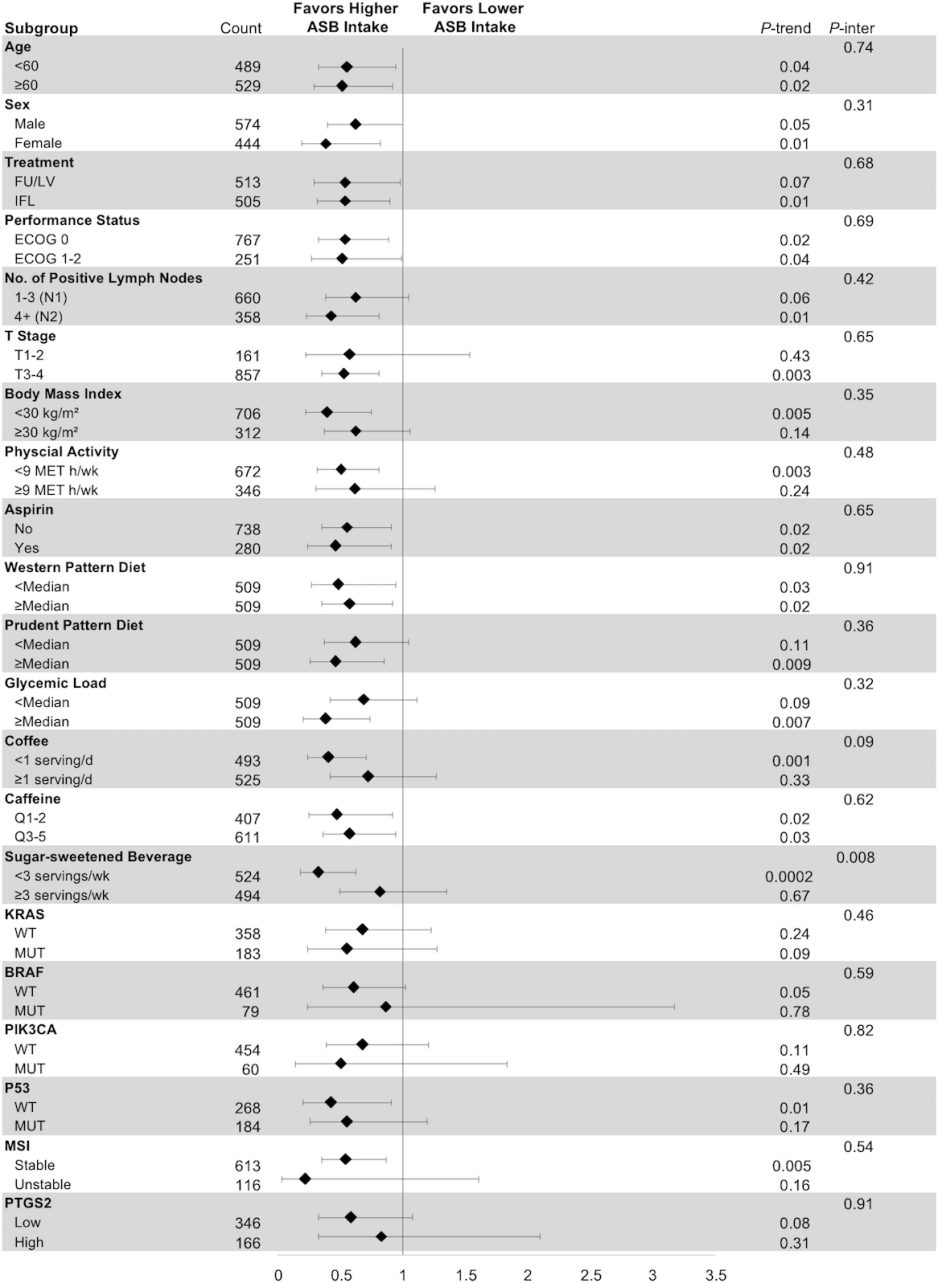
Multivariable hazard ratios (HRs) and 95% confidence intervals (CIs) for cancer recurrence or mortality across strata of patient demographics, treatment and disease characteristics, and dietary and lifestyle factors. Analyses used four categories of artificially sweetened beverage intake (<2 servings per month, 2 per month to 2 per week, 3 to 6 per week, and ≥1 servings per day). The forest plot represents the HRs of the comparison of 1 serving per day or more of artificially sweetened beverages with less than 2 servings per month, adjusting for age (continuous variable in years), sex (male or female), depth of invasion through bowel wall (binary variable, pT1-2 or pT3-4), number of positive lymph nodes (binary variable, 1–3 nodes or ≥4 nodes), baseline performance status (binary variable, 0 or 1–2), chemotherapy treatment group (binary variable, 5-fluorouracil and leucovorin or irinotecan, 5-fluorouracil, and leucovorin), consistent aspirin use (yes on Q1 and Q2), and time-varying total calorie intake (continuous variable, in kilocalories per day), physical activity (continuous variable in metabolic equivalent task hours per week), and body mass index (continuous variable in kilogram per meter-squared). *P* values are two-sided; p-inter indicates *P* for interaction between strata and artificially sweetened beverage intake; p-trend indicates *P* for trend across levels of artificially sweetened beverage intake. Abbreviations: BMI, body mass index; d, day; FU/LV, fluorouracil and leucovorin; IFL, irinotecan, fluorouracil, leucovorin; kg/m^2^, kilogram per meter-squared; MET h/w, metabolic equivalent task hours per week; MSI, microsatellite instability; MUT, mutant; No., number; Q1, questionnaire 1, midway through adjuvant chemotherapy; Q2, questionnaire 2, 14 months after surgery; Q1-2, quintiles 1 and 2; Q3-5, quintiles 3 to 5; p-inter, *P* for interaction; T stage, tumor stage; wk, week; WT, wild type.

We examined the influence of artificially sweetened beverage intake on DFS across strata of other potential predictors of patient outcome, comparing ≥1 12-oz. servings per day to <2 servings per month (**Fig 2**). The association between artificially sweetened beverage intake and patient outcome appeared consistent across most strata of patient, disease, and treatment characteristics. We further tested other exposures associated with energy excess, demonstrating similarities across strata. Additionally, we showed similar associations regardless of molecular tumor characteristics. Tests for interaction between artificially sweetened beverage intake and the examined covariates—including molecular covariates such as tumor *KRAS*, *P53*, and MSI status—were non-significant except for sugar-sweetened beverage intake. The association of artificially-sweetened beverage intake with improved DFS appeared stronger among patients consuming <3 sugar-sweetened beverages per week compared to individuals with more frequent sugar-sweetened beverage intake (*P*_interaction_ = .008).

### Substitution analysis

**Fig 3:**
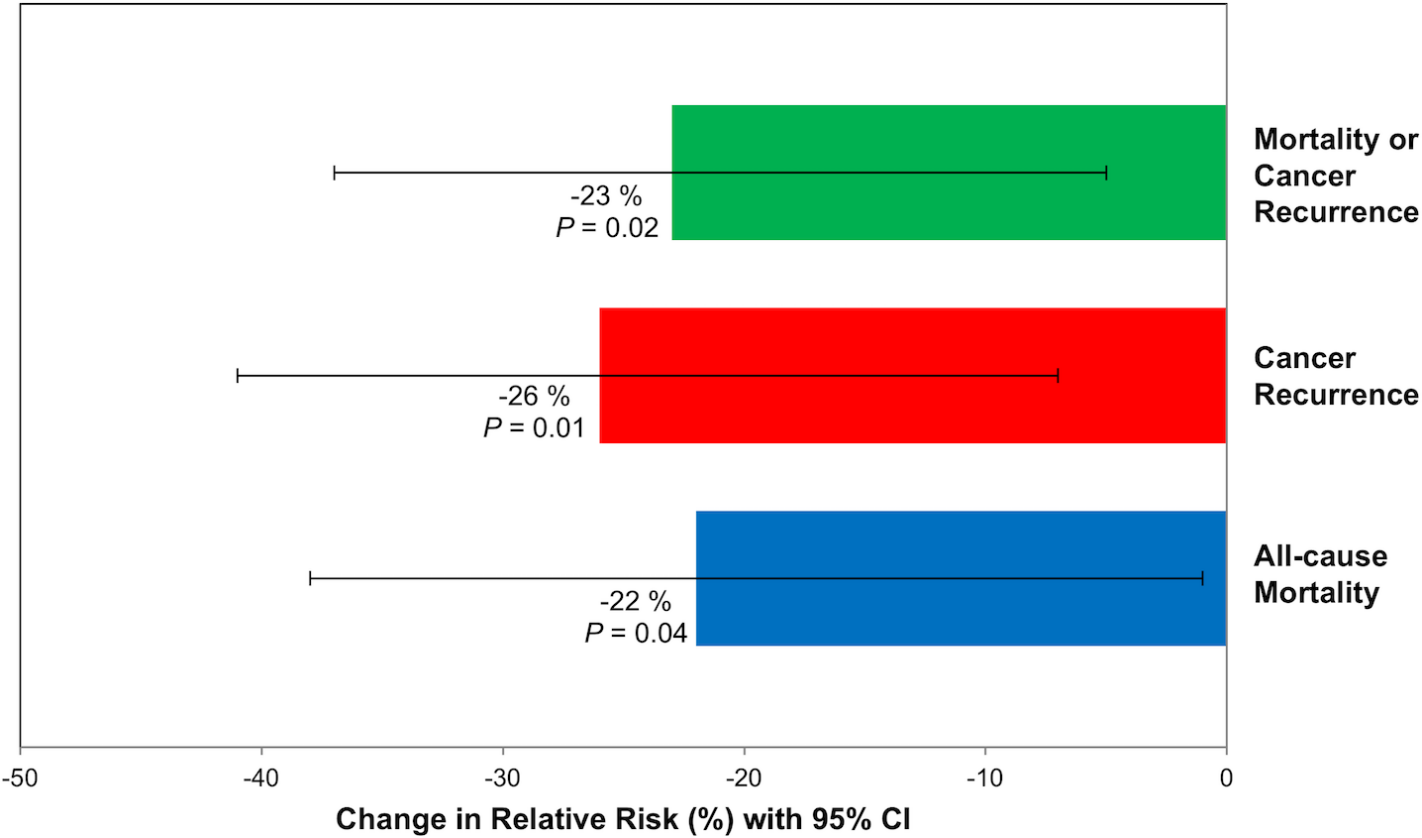
Results of isovolumetric substitution analysis, calculating change in relative risk of patient outcomes with substitution of 1 serving/day of artificially sweetened beverage for sugar-sweetened beverage. Change in relative risk is displayed for each outcome as a percentage both graphically and in text, with 95% confidence intervals depicted graphically as error bars. Adjusted for age (continuous variable in years), sex (male or female), depth of invasion through bowel wall (binary variable, pT1-2 or pT3-4), number of positive lymph nodes (binary variable, 1–3 nodes or ≥4 nodes), baseline performance status (binary variable, 0 or 1–2), chemotherapy treatment group (binary variable, 5-fluorouracil and leucovorin or irinotecan, 5-fluorouracil, and leucovorin), consistent aspirin use (yes on both questionnaire 1 and 2), and time-varying total calorie intake (continuous variable, in kilocalories per day), physical activity (continuous variable in metabolic equivalent task hours per week), and body mass index (continuous variable in kilogram per meter-squared). *P* values are two-sided. Abbreviations: CI, confidence interval.

We sought to estimate the influence of substituting artificially sweetened beverages for sugar-sweetened beverages on patient outcome (**Fig** 3**)**. In substitution analyses, replacing one 12-oz. serving of a sugar-sweetened beverage per day with an isovolumetric serving of an artificially sweetened beverage was associated with a significant improvement in patient outcomes: 23% for DFS (HR, 0.77; 95% CI, 0.63 to 0.95, *P* = .02), 26% for RFS (HR, 0.74; 95% CI, 0.59 to 0.93, *P* = .01), and 22% for OS (HR, 0.78; 95% CI, 0.62 to 0.99, *P* = .04).

## Discussion

In this prospective study of stage III colon cancer patients enrolled in a NCI-sponsored clinical trial, increasing artificially sweetened beverage intake was associated with a significant improvement in DFS, RFS, and OS. These associations persisted after adjusting for potential confounders, including measures of energy balance such as BMI, physical activity, Western pattern diet and dietary glycemic load. Isovolumetric substitution of artificially sweetened beverages for sugar-sweetened beverages was associated with a lower risk of mortality and cancer recurrence, suggesting that these findings may be accounted for, at least in part, by patients consuming artificially sweetened beverages instead of sugar-sweetened beverages.

To our knowledge, this is the first study of artificially sweetened beverage intake and colon cancer recurrence and survival. In light of studies indicating increased risk of colon cancer recurrence and mortality in association with higher sugar-sweetened beverage intake and other states of energy excess (e.g., sedentary lifestyle, high dietary glycemic load, Western pattern diet, elevated plasma C-peptide), we hypothesized that artificially sweetened beverage intake might reduce colon cancer recurrence by substituting for and thereby reducing intake of high-calorie, sugar-sweetened alternatives [2, 4–7, 15]. The impact of artificially sweetened beverage intake on long-term energy balance, as denoted by conditions such as obesity and type 2 diabetes, remains controversial; while experimental studies generally suggest that substituting artificially sweetened for sugar-sweetened beverages reduces energy excess [20, 29, 55–60], large observational studies have produced mixed results [14, 20, 22–28]. This may be due in part to reverse causality, as individuals in observational studies at highest risk for weight gain may choose to increase artificially sweetened beverage consumption in attempts at weight control [20, 26, 61–63]. In contrast, studies have more consistently linked increased sugar-sweetened beverage consumption to incidence of type 2 diabetes and obesity [10–14], which have in turn been associated with worsened colon cancer patient outcomes [6, 64]. Of note, all patients in this study received surgical resection followed by adjuvant chemotherapy; therefore, our findings do not suggest that any dietary habit should substitute for standard surgery or adjuvant chemotherapy.

As we have described previously,[53] examining relationships between dietary factors and cancer patient outcome in a NCI-sponsored randomized clinical trial provides several advantages. In the trial, patient follow-up and treatment were carefully standardized and included regular detailed medical examinations to prospectively document cancer recurrence. Prospective measurement of potentially prognostic variables at study enrollment also reduced likelihood of reporting bias, facilitating more accurate adjustment for potential confounders. Finally, all patients had lymph node-positive cancer without distant metastasis at trial enrollment, reducing the influence of confounding by disease stage.

Given our study’s observational design, we cannot completely exclude the possibility of residual confounding. However, the observed associations persisted after adjusting for known and suspected predictors of patient outcome. Furthermore, the association between artificially sweetened beverage intake and improved DFS remained largely consistent across strata of these potential confounders. Nonetheless, other potential confounders plausibly associated with artificially sweetened beverage intake, such as socioeconomic status [61], were not assessed in our cohort.

We considered the possibility that patients with occult cancer recurrences or other poor prognostic characteristics may have decreased artificially sweetened beverage intake due to poor appetite or other cause. To minimize this bias, we excluded individuals who experienced recurrence or death within 90 days following Q1 completion. When we extended this restriction to 180 days, individuals reporting consumption of ≥1 artificially sweetened beverages per day continued to demonstrate lower risk of mortality or disease recurrence. Furthermore, we would expect few patients to have occult cancer recurrences or other poor prognostic characteristics at baseline because patients underwent comprehensive clinical and radiologic assessment before trial enrollment. To account for change in diet over time—including dietary changes following recovery from treatment—and to mitigate random error in reporting of dietary practices through repeated measures, we updated dietary data with a second FFQ 6 months following completion of adjuvant chemotherapy. Given the likelihood that individuals who consume artificially sweetened beverages following cancer diagnosis also consumed artificially sweetened beverages prior to cancer diagnosis, we cannot exclude the possibility that individuals who consume artificially sweetened beverages develop biologically less aggressive colon cancer tumors. Future studies should examine artificially sweetened beverage intake both before and after colon cancer diagnosis in order to further elucidate the relationship between artificially sweetened beverage intake and colon cancer recurrence and mortality. In addition, we note the number of patients in our study consuming ≥2 servings of artificially sweetened beverages daily was relatively small (n = 42), resulting in 95% confidence intervals for this category crossing one. Despite this, the statistical tests for trend across categories of artificially sweetened beverage intake was statistically significant and the 95% confidence intervals did not cross one after collapsing the two highest frequency categories of artificially sweetened beverage consumption to improve statistical power. Nonetheless, the lack of precision indicated by the confidence intervals indicate that replication of our analyses in other cohorts is required to confirm our findings. We also note that the results of our secondary analyses should be considered exploratory and hypothesis generating.

Finally, randomized trials may select patients who differ from the general population. However, the pattern of dietary and lifestyle habits reported by our cohort were consistent with those of other U.S. cohorts [65]. Moreover, this large trial enrolled patients from community and academic centers throughout North America, reducing likelihood of biased sampling.

In sum, this prospective study of patients with stage III colon cancer, embedded in a randomized clinical trial, suggests improved patient outcome with increased consumption of artificially sweetened beverage, which may be accounted for, completely or in part, by substitution for sugar-sweetened alternatives. Although our study cannot offer evidence for causality given its observational design, our findings offer further insight into the role of diet and lifestyle in colon cancer outcomes and potentially meaningful recommendations for clinical care. Further studies are needed to confirm these findings.

## Supporting information

S1 File SAS log files for statistical analyses. (LOG)
